# Effects of exposure to fine particulate matter in elderly hospitalizations due to respiratory diseases in the South of the Brazilian Amazon

**DOI:** 10.1590/1414-431X20188130

**Published:** 2019-01-24

**Authors:** A.B. Machin, L.F. Nascimento, K. Mantovani, E.B. Machin

**Affiliations:** 1 Faculdade de Engenharia de Guaratinguetá, Universidade Estadual Paulista Júlio de Mesquita Filho, Guaratinguetá, SP, Brasil; 2 Programa de Pós-Graduação em Ciências Ambientais, Universidade de Taubaté, Taubaté, SP, Brasil; 3 Faculdade de Tecnologia de Guaratinguetá (FATEC), Guaratinguetá, SP, Brasil; 4 Departamento de Ingeniería Mecánica, Facultad de Ingeniería, Universidad de Concepción, Concepción, Chile

**Keywords:** Air pollutants, Particulate matter, Respiratory diseases, Elderly health, Mathematical modeling

## Abstract

Exposure to air pollution is an important cause of hospital admissions due to respiratory diseases. Nevertheless, few studies use pollutant concentration data estimated by mathematical models. A time-series ecological study was developed, using data from hospitalizations due to respiratory diseases in people over 60 years of age, residents of Cuiabá, Brazil, during 2012, obtained from the Brazilian Ministry of Health. The independent variables were the concentrations of fine particulate matter (PM_2.5_) and carbon monoxide (CO) estimated by mathematical modeling, minimum temperature, and relative humidity (obtained from the Brazilian Meteorological Agency), and the number of forest fires. The generalized linear regression model of Poisson was used, with lags of 0 to 7 days. The coefficients obtained were transformed into relative risk of hospitalization, with respective 95% confidence intervals; alpha=5% was adopted. In that year, 591 hospitalizations were evaluated, with a daily average of 1.61 (SD=1.49), the PM_2.5_ average concentration was 15.7 µg/m^3^, and the CO average concentration was 144.2 ppb. Significant associations between exposure to these contaminants and hospitalizations in lags 3 and 4 in 2012 were observed. There was a hospitalization risk increase of 31.8%, with an increase of 3.5 µg/m^3^ of PM_2.5_ concentrations and an increase of 188 in the total number of hospitalizations, with an expense of more than ≈US$ 96,000 for the Brazilian Public Health System. This study provided information on the cost of air pollution to the health system and the feasibility of using a mathematical model to estimate environmental concentration of air pollutants.

## Introduction

Respiratory diseases, diagnosed according to Chapter X of the International Classification of Diseases, 10th Revision (ICD-10), were responsible for almost 323,000 hospitalizations of the people over 60 years of age in Brazil in 2012 generating costs for the Brazilian Public Health System (SUS) above R$ 293,000,000 (≈US$ 150,000,000). More than 5,000 hospitalizations occurred in Mato Grosso state, generating costs close to R$ 4,200,000 (≈US$ 2,200,000); 591 hospitalizations were registered only in Cuiabá, MT, generating expenses to SUS above the R$ 590,000, ≈US$ 302,000 (1).

Recent studies have evaluated the adverse effects of air pollutants on the health of the population, including mortality rates, hospital admissions, and emergency room visits in hospitals because of cardiovascular, respiratory, and other diseases (2–9). Other studies have shown that high levels of air pollution increase morbidity and mortality rates (10).

Particulate matter (PM) is a mixture of liquid and solid particles suspended in air, of which composition and size depend on the emission sources. These particles are classified into two groups: particles with a diameter between 2.5 and 10 μm called coarse mode and particles with a diameter of less than 2.5 μm called fine particulate that represent about 60 to 70% of the amount of PM_10_ (11,12). Carbon monoxide has an affinity for hemoglobin 240 times greater than oxygen, which causes a small amount of CO to saturate a large number of hemoglobin molecules, reducing the ability of the blood to carry O_2_. It also acts by diverting the dissociation curve of hemoglobin to the left leading to a decrease in O_2_ release in tissues. Automotive vehicles, industrial processes, biomass burning, among others, are pointed to as the main sources of emission of particulate matter and CO to the atmosphere, according to the World Health Organization (13).

These pollutants are usually quantified by measuring stations of state environmental agencies. Nevertheless, not all states have environmental agencies, which can use mathematical models to estimate concentrations of air pollutants. An example of this model is Coupled Chemistry Aerosol and Tracer Transport model of the Brazilian developments in the Regional Atmospheric Modeling System (CCATT-BRAMS) (14,15), already validated by Longo et al. (15). This model has been used operationally by the Center for Weather Forecasting and Climate Studies of the National Institute for Space Research (CPTEC/INPE) (16) and was used in previous studies (17–20).

The objective of this study was to identify the effects of exposure to fine particulate matter and CO on the number of hospitalizations due to respiratory diseases in elderly people residing in Cuiabá, Mato Grosso, a Brazilian state that does not have measuring stations of environmental agencies. The CCATT-BRAMS mathematical model estimated the values for the development of this study.

## Material and Methods

An ecological time-series study was developed, with data related to hospitalizations due to respiratory diseases, such as pneumonia (J12.0–J18.9), bronchitis, and bronchiolitis (J20.0–J21.9), chronic obstructive pulmonary disease (J44.0–J44.9), and asthma (J45.0–J45.9), from ICD-10, in people of 60 years or older residing in Cuiabá, MT, Brazil. The values were obtained from the Department of Informatics of the National Health System (Datasus) (1). The studied period was from January 1, 2012 to December 31, 2012.

**Figure 1:**
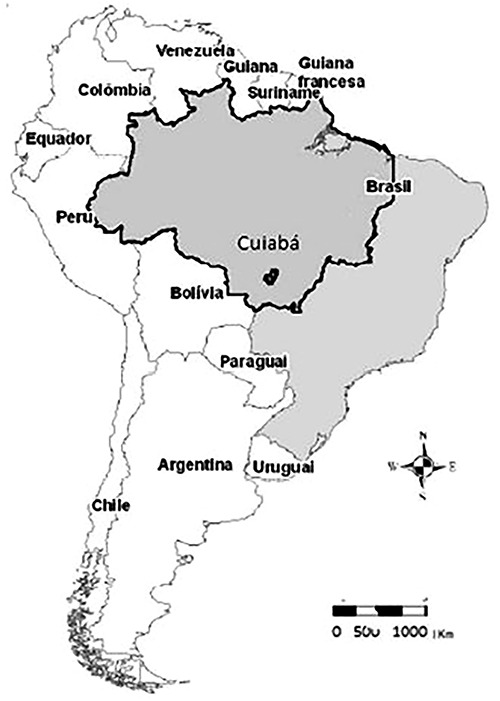
Geographic location of the municipality of Cuiabá, Mato Grosso state, Brazil.

Cuiabá is the capital city of Mato Grosso state, with a population of approximately 600,000 inhabitants. It is located in the center of South America, at 15°36’ S and 56°06' W, so the climate is tropical (Figure 1). The rainy season is from October to April, the climate of the rest of the year is very dry. Cold fronts inhibit cloud formations, which causes constant forest fires. This city has a Human Development Index (HDI) of 0.785 and has 17 private hospitals and 11 hospitals that provide care for the SUS with about 1400 beds for hospitalization (21).

According to National Institute of Meteorology (INMET) (22), in the warmer month, the temperature can reach 40°C with average of 26°C and in the winter, the temperature can drop below 10°C due to the cold fronts coming from the south of the continent.

Pollution levels in Cuiabá are mainly determined by emissions from local industries, as well as a large number of forest fires recorded per year and the large fleet of vehicles over 400,000 (23).

CCATT-BRAMS is a mathematical model that allows numerical simulations of weather and climate, solving large phenomena explicitly on spatial scales and by parameterizing the processes that occur at scales smaller than the spatial resolution of the model. The Center for Weather Forecasting and Climate Research of the National Institute of Space Research (CPTEC-INPE) does this modeling process on a daily basis, producing daily diagnoses and predictions for up to three days, covering all of South America. The model takes into consideration transportation of various gases and aerosol particles, such as carbon monoxide (CO) and fine particulate matter (PM_2.5_), which are estimated from the number and locations of fires outbreaks that are observed through remote sensors, thus generating daily estimates of various pollutants. The horizontal resolution of this operation is 25 km by 25 km, with 38 atmospheric levels, of which the first level is from ground level to 40 m above the ground, and this method has already been validated. These records are estimated daily every 3 h (14,15).

The number of hospitalizations was obtained for the months of January and February of 2013 to identify the cases of November and December of 2012 that were reported in later dates.

The daily number of hospital admissions due to respiratory diseases was considered the dependent variable, the mean daily concentrations of PM_2.5_ and CO were estimated by the CCATT-BRAMS model and were considered the independent variables. The days of the week, holidays, number of days elapsed since the beginning of the period, daily mean temperature, daily mean relative air humidity, and forest fires, which may interfere directly or indirectly with respiratory diseases, were introduced as control variables in the models.

The daily data for forest fires were obtained from the Environmental Information System (SISAM), and information about air humidity and temperature was obtained from the INMET (22) in Cuiabá. The daily averages of PM_2.5_ pollutants and CO were calculated, being quantified in μg/m^3^ and ppb, respectively.

As the effects of exposure to pollutants can be noticed on the same day and subsequent days, a lag of zero to seven days (lag 0–lag 7) was used since there is no consensus on the extent of this period.

This analysis used the generalized linear model of Poisson regression. The coefficients provided by the model were transformed into relative risk (RR) of hospitalization according to the expression:

(Eq. 1) RR=exp(β*Conc)

where β is the coefficient obtained by Poisson regression and *Conc* is the pollutant concentration. The confidence interval (95% CI) for RR was also calculated.

The risk of hospitalization and the percentage increase of the risk was estimated according to the increase observed in the interquartile difference (IQD) between the 75th and 25th percentiles. The percentage change in risk was calculated by:

(Eq. 2) ∆RR=[exp(β*IQD)−1]*100

With the values of RR according to IQD in the PM_2.5_ concentration, the proportional attributable ratio (PAR) was calculated using the following formula:

(Eq. 3) PAR=1−1/RR

The PAR value shows the percentage increase in the risk of hospitalization and allows to estimate the number of hospitalizations associated with this increase through the population attributable fraction (PAF) obtained by the formula:

(Eq. 4) PAF=PAR*N

where *N* is the number of hospitalizations of elderly patients with respiratory tract diseases, during the studied period.

After calculating the PAF, and knowing the average cost of the hospitalizations in the year (≈US$ 500), it was possible to calculate the costs for the Public Health System that could be avoided with a decrease of IQD in the concentration of PM_2.5_. All analyses were performed using the statistical program Statistica v.7 (StatSoft, USA). The level of significance adopted in this study was 5%.

This study was not submitted to an Internal Review Board (Ethics Committee) because the records are available in Datasus, a public website.

## Results

During the study period, there were 591 hospitalizations of elderly due to respiratory diseases. The mean daily hospitalization was 1.61 (SD=1.49) and ranged from 0 to 7 admissions. The mean concentration of PM_2.5_ was 15.7 μg/m^3^ (SD=3.2) and CO was 144.2 ppb (SD=52.3) (Table 1).

**Table 1:** Concentration values of particulate matter (PM_2.5_), CO, minimum temperatures, relative humidity and number of hospitalizations in Cuiabá, Brazil, 2012.

	Mean (SE)	Min–Max
Hospitalizations	1.6 (1.5)	0–7
PM_2.5_ (µg/m^3^)	15.7 (3.2)	12.0–28.3
CO (ppb)	144.2 (52.3)	69.0–317.1
Minimum temperature	20.6 (3.1)	9.0–27.8
Relative humidity (%)	70.4 (13.7)	35.0–96.0

There were 20 days without values for PM_2.5_ concentration (5.5% of the analyzed period). In addition, in 9 days (2.5%), the mean concentrations of PM_2.5_ presented values above the limit considered tolerable (25 μg/m^3^).

Exposure to fine particulate material was significantly associated (P<0.05) with hospitalizations due to respiratory diseases three and four days after exposure (lag 3 and lag 4) (Table 2).

**Table 2:** Coefficients with the respective standard errors provided by the Poisson regression model after 0 to 7 days of exposure to particulate matter PM_2.5_) pollutant and hospitalizations in Cuiabá, Brazil, 2012.

Lags	Coefficient	SE
Lag 0	0.0567	0.0361
Lag 1	–0.0053	0.0366
Lag 2	0.0530	0.0369
Lag 3^*^	0.0846	0.0380
Lag 4^*^	0.1095	0.0368
Lag 5	0.0139	0.0382
Lag 6	0.0441	0.0380
Lag 7	–0.0355	0.0370

^*^P<0.05

**Figure 2:**
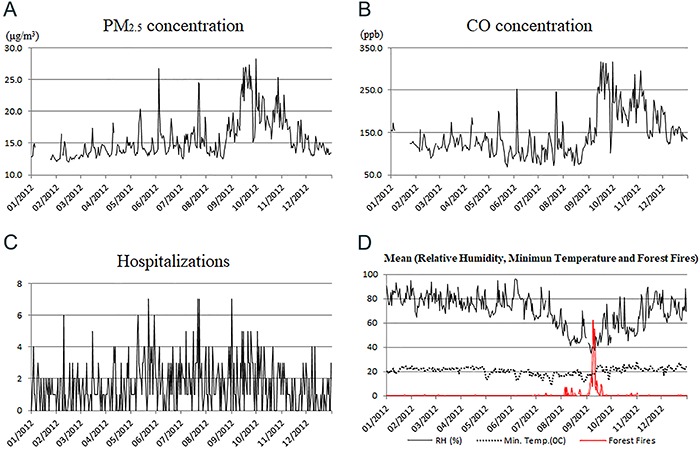
Temporal distribution of particulate matter (PM_2.5_) (**A**); CO concentration (**B**); hospitalizations (**C**); minimum temperature, air relative humidity (RH), and forest fires (**D**), in Cuiabá, MT, Brazil, 2012.

Figures 2A to 2D show the distribution of concentrations of fine particulate matter, CO, daily number of hospitalizations, minimum temperature, and relative humidity of air in 2012. The largest concentrations of PM_2.5_ and CO were in the second half of the year, when temperatures, relative humidity, and rainfall are very low, and 98.01% of recorded fires occurred during this period, which potentiates the risk of hospitalizations.

The increase observed in the IQD between the 75th and 25th percentiles was 3.5 μg/m^3^ for PM_2.5_.

**Figure 3:**
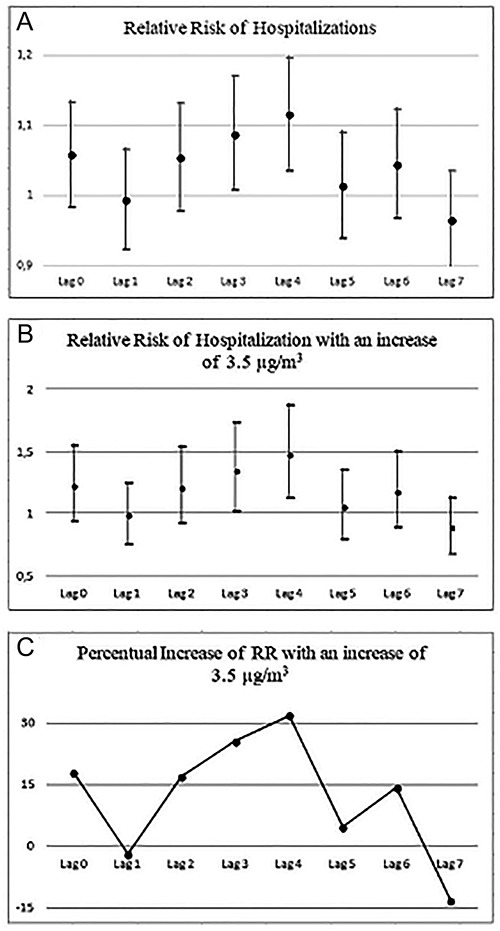
Relative risk (RR) of hospitalization after 0 to 7 days of exposure to particulate matter (PM_2.5_) (**A**), with an increase of 3.5 µg/m^3^ (**B**), and RR percentage increase (**C**) in Cuiabá, MT, Brazil, 2012.

The relative risk of hospitalization after exposure to PM_2.5_ (Figure 3A), with an increase of 3.5 μg/m^3^ (Figure 3B), and percentage increase in relative risk (Figure 3C) were recorded. A significant association between exposure to PM_2.5_ and hospitalizations on the third day (lag 3, RR=1.09) and the fourth day (lag 4, RR=1.12) after exposure was found for the studied period, which allows a real view of the influence of air pollutant exposure on health outcomes.

Figure 3C shows the percentage increase of RR of hospitalization and lag 3 and lag 4 were associated with hospitalizations, increasing the risk by 25.6% and 31.8%, respectively. The increase of 3.5 μg/m^3^ in the PM_2.5_ concentration was used to calculate the number of additional hospitalizations, representing an increase of 188 elderly hospitalizations, which considering the average hospitalization daily value of R$ 998.71, would increase the costs to the Public Health System by approximately R$ 188,000 (≈US$ 96,000). This indicates the importance of developing public policies to reduce the risks of pollution to the health of the population, especially of the elderly.

## Discussion

There are few studies carried out in Cuiabá about the effects of air pollutants on hospitalizations of elderly of 60 years or older due to respiratory diseases. This study identified a significant association with exposure to PM_2.5_ in lags 3 and 4 and this increase would entail an increase in the cost for the SUS of approximately US$ 96,000.

Another study carried out in Cuiabá in 2005 used this kind of analysis with data estimated by the CCATT-BRAMS and found an association between exposure to PM_2.5_ and hospitalizations due to respiratory diseases in children under 5 years of age in lags 1, 2, and 5 for the whole year, and in the lags 1, 5, and 6 in the dry period (second semester), as a function of a 10 μg/m^3^ increase. Nevertheless, no association was found among elderly people over 60 years in the studied period. The daily mean annual and second semester values of PM_2.5_ concentration of that study were 7.5 μg/m^3^ and 11.9 μg/m^3^, respectively, which were lower than the values calculated in this study (15.67 μg/m^3^ and 17.03 μg/m^3^) (18). Those authors concluded that particulate emissions from forest fires in the Amazon are related to the prevalence of hospitalizations due to respiratory diseases in sensitive population groups in the municipalities of Mato Grosso State. (18)

Some studies published in Brazil point to forest fires as responsible for health problems (24–27). The increase in the number of fires in the dry season of the year leads to significant increases in the levels of air pollutants, which translates into an increase in the number of hospitalizations due to respiratory diseases.

An ecological and exploratory study was conducted using data from the state of Mato Grosso from 2008 and 2009 on hospital admissions of children aged 0 to 4 years due to pneumonia and on fires in the same period. That study identified municipalities that required interventions to reduce rates of admission due to pneumonia and the number fires (26).

A time-series ecological study of children and elderly hospitalizations due to respiratory diseases was carried out in Alta Floresta and Tangará da Serra, Brazilian Amazon, using estimates of daily concentrations of PM_2.5_ resulting from the burning of biomass, and meteorological and calendar variables. The study identified significant increases in the relative risk of hospitalizations due to respiratory diseases in children for the whole year and for the dry period, with Lags of 3–4. For the elderly, the increase was significant for the current day in the dry season, with a 6.8% increase (95%CI: 0.5–13.5) with a 10 μg/m^3^ increase of PM_2.5_ (24). Indicators of atmospheric pollution showed an association with respiratory disease occurrences in the Brazilian Amazon region, especially in the more vulnerable age groups, which may be used to assess the effects of forest fires on human health (25).

Another study related the effects of mean daily concentration and maximum hourly concentration of air pollution of fine particles (PM_2.5_) with mortality in six cities of the Pearl River Delta in China (28). A significant association between the hourly peak of PM_2.5_ and mortality was identified.

Studies have shown that decreases in PM_2.5_ concentrations represent significant decreases in the number of hospitalizations and deaths due to respiratory, cardiovascular, and other diseases, as well as reductions in Public Health System costs (29). The concentrations of NO_2_ allow a better evaluation of the effects of air pollutants on respiratory health than the PM_10_ particles (30). In São José dos Campos, SP, a Brazilian city of medium size, the accumulated effect of eight days showed that for 24.7 μg/m^3^ increases in the mean concentration of PM_10_ there was an increase of 9.8% in hospitalizations (31). Other studies have shown that air pollution levels, generally represented by PM_10_, PM_2.5_, NO_2_, SO_2_, and O_3_ rates, are associated with the short-term increase in emergency room attendances due to respiratory problems (32–35).

Another study analyzed deaths due to respiratory diseases considering differential susceptibility according to sex using Poisson regression and identified an increase in the risk of death with exposure to PM_10_ (36).

Exposure to nitrogen oxides (NOx) emitted by burning fossil fuels was associated with deaths caused by respiratory diseases using data estimated by the CCATT-BRAMS. Exposure to NOx was significantly associated with mortality owing to respiratory diseases: relative risk (RR)=1.035 for lag 2, RR=1.064 for lag 3, RR=1.055 for lag 4, and RR=1.042 for lag 5 (19).

In addition, a study carried out in Taubaté, Brazil, identified only NOx to be associated with hospitalizations due to respiratory diseases, evidenced in lags 1 and 4, and the RRs for hospitalization were 1.046 (95%CI: 1.015–1.079) and 1.054 (95%CI: 1.054–1.196), respectively (20). Nevertheless, César et al. (17) studied the possible association between exposure to PM_2.5_ and hospitalizations due to pneumonia and asthma in the same period, but in children up to 10 years of age, and the relative risk of hospitalization values were significant for lags 0 and 2–5. A 20.3 to 38.4% increase in hospitalization risk was observed when the concentration of PM_2.5_ was increased by 5 μg/m^3^, resulting in an increase of 38 hospitalizations.

This study may have some limitations; one of them may lie in the fact that fine particulate was estimated by mathematical modeling. Also, the force of the winds at the study site was not considered, which could dilute or increase the concentration of pollutants, bringing pollutants from regions near Cuiabá, which could alter the number of hospitalizations due to respiratory diseases. Another possible limitation is that, although data from hospitalizations were obtained from an official source (Datasus), it can contain diagnostic errors. In addition, it does not provide information on the nutritional status of the elderly, their medical history, living conditions, smoking status, among others that may be associated with respiratory diseases. The hospitalization data refers only to those occurring in the public network, excluding private hospitalizations, those from health plans, or health operators. Our findings should be used mainly for accounting purposes as it is not possible to establish a cause and effect relationship between exposure to pollutants and hospitalizations. However, an association between pollution and hospitalizations due to respiratory diseases was found, where exposure to PM_2.5_ and CO was statistically significant and may be considered a risk factor for respiratory diseases. It is important to emphasize that the study indicates an association rather than causality.

It was possible to find a statistically significant association between exposure to PM_2.5_ and hospitalizations due to respiratory diseases. It is also important to highlight the importance of using the CCATT-BRAMS model, which allowed the collection of pollutant concentration data in a region without a measuring station. The results presented should influence the development of public policies for the reduction of pollutants in the air, reducing costs for the SUS, as well as improving the quality of life of the general population.
